# Web-based treatment for depression in pregnancy: a feasibility study of Mum2BMoodBooster

**DOI:** 10.1186/s12888-022-04111-x

**Published:** 2022-07-16

**Authors:** Alan W. Gemmill, Jessica Lee Oliva, Jennifer Ericksen, Charlene Holt, Christopher J. Holt, Jeannette Milgrom

**Affiliations:** 1 Parent-Infant Research Institute, Heidelberg Repatriation Hospital, Heidelberg Heights, VIC 3081 Australia; 2 Department of Psychology, College of Health Care Science, James Cook University, Townsville, QLD Australia; 3 Australian College of Applied Professions, Melbourne, VIC 3000 Australia; 4 Melbourne School of Psychological Sciences, University of Melbourne, VIC, 3010 Australia

**Keywords:** Antenatal depression, Internet intervention, Web-based intervention, Feasibility trial, cognitive-behavioural therapy

## Abstract

**Background:**

Depression in pregnancy is prevalent, under-treated, and has serious impacts on the wellbeing of women and on child development. Internet programs can reach women who may not access traditional treatments due to distance, stigma or concern about taking medication. We adapted our online postnatal depression program, *MumMoodBooster*, for antenatal use. We aimed to trial feasibility, acceptability, and potential efficacy of the new *Mum2BMoodBooster* intervention with depressed pregnant women.

**Methods:**

Twenty-seven pregnant women with Edinburgh Postnatal Depression Scale score > 11 used the program in a feasibility trial. Twenty-one had current diagnoses of major or minor depression on the Structured Clinical Interview for the DSM-IV. Assessment of symptoms occurred at screening/baseline, post-test (8 weeks post-enrollment), and at follow-up (3 months postpartum) using the Patient Health Questionnaire (PHQ-9) and the Depression Anxiety Stress Scales (DASS-21).

**Results:**

In this feasibility trial, depression scores on both the PHQ-9 and the DASS-21, showed significant reductions representing large effects, with average symptom scores reduced by > 50%, and maintained in the ‘minimal or no depression’ range at 3 month follow-up. Anxiety scores also decreased significantly. Program usage was high with 74% of women visiting all six sessions. Program acceptability ratings were moderate to high.

**Conclusions:**

Findings paralleled the magnitude of symptom reductions seen in randomised trials of the postnatal *MumMoodBooster* program, suggesting that *Mum2BMoodBooster* is an effective treatment for depressed pregnant women. Effective internet therapies are likely to become increasingly important as the COVID-19 pandemic continues to make face-to-face access to health care problematic during ‘lockdowns’.

## Introduction

Depression during pregnancy (antenatal depression) is common [1]. A review of studies in 14 developed countries reported a pooled prevalence of 17% [2], which is broadly consistent with previous meta-analyses based on diagnostic criteria [3] and recent cross-sectional studies [4]. Antenatal depression is also a significant risk factor for postnatal depression [5].

In addition, children born to mothers who experienced antenatal depression are more likely to have behavioural and emotional problems. This includes an increased risk of anxiety and attention deficit/hyperactivity in childhood [6–10], and increased risk of depression [11] and anxiety disorders [12]. Negative effects, of sizes that are clinically important, have been found to endure at least to adolescence [11, 12].

A range of treatment approaches are effective for perinatal depression. Systematic reviews by Dennis and Hodnett, and by Cuijpers and colleagues [13, 14] broadly confirm the efficacy of pharmacotherapy, cognitive behavioural therapy (CBT), counselling, and interpersonal psychotherapy [13, 14].

Despite the availability of clinic-based treatments, fewer than 50% of depressed perinatal women are identified and even fewer seek help [15]. Internet-based interventions have potential to overcome many barriers to help-seeking by providing privacy, anonymity and ready access from home. Internet-based interventions for perinatal mental health difficulties (e.g., [16–18]) have been shown in meta-analyses to achieve medium-sized effects in reducing depressive symptoms [19, 20]. However, there are very few published studies that have examined internet-based cognitive behavioural therapy (CBT) interventions, specifically for diagnosed antenatal depression [21].

We previously developed a six-session online CBT program for women with postnatal depression, *MumMoodBooster* [22], based on Milgrom’s *Getting Ahead of Postnatal Depression* program [23, 24]. The program, supported by weekly calls from a coach, was evaluated in a feasibility trial [25] and two randomised controlled trials (RCT) [16, 26].

In the first RCT, all women met the Diagnostic and Statistical Manual of Mental Disorders-IV (DSM-IV) criteria for major or minor depression at enrolment [16]. Following MumMoodBooster, 79% no longer met these criteria, compared to only 18% remission in the treatment as usual condition—a fourfold improvement in remission from a DSM-diagnosed depressive disorder. The second RCT found a similar pattern, and demonstrated an efficacy at least as good as specialised face-to-face CBT treatment [26].

Given the efficacy of the postnatal *MumMoodBooster* program, we adapted the core components for antenatal women. The aim of this study was to evaluate the new *Mum2BMoodBooster* program in a feasibility trial. It was hypothesised that the program would be well accepted, show similar adherence to the postnatal program and demonstrate improved mood ratings over time similar to those seen in RCTs of *MumMoodBooster*.

## Methods

The research was approved by the Human Research Ethics Committee of Austin Health (approval number HREC/16/Austin/339). All participants provided written informed consent.

### Adaptation of MumMoodBooster to Mum2BMoodBooster

The postnatal *MumMoodBooster* program comprises six interactive sessions that are sequentially accessed weekly, designed to parallel face-to-face treatment [22]. The sessions utilize cognitive-behavioural therapy techniques to target depression and comorbid anxiety, including mood monitoring, relaxation training, behavioural activation, and strategies for managing cognitive distortions associated with depression and anxiety. Each session begins with a video introducing content. Sessions include text, animations, videos, tutorials, and case vignettes relevant to life in the postpartum period. Personalised content such as lists of pleasant activities and goals are created within the program. Self-monitoring tools, such as daily mood and activity tracking, are depicted in charts. Homework activities are to be completed between sessions. Users have access to library articles on topics including stress management, problem solving, parenting support etc., as well as a partner support website that mothers can invite their partner to access. Low intensity (< 30 min) telephone coaching once a week encourages participants to use the program.

The content of the *MumMoodBooster* program was modified to be relevant for an antenatal population. Session welcome videos and several video vignettes were edited to remove postnatal-specific content and five new antenatal vignettes were recorded. Text was changed to be specifically relevant to antenatal women, and images of antenatal women replaced postnatal images. Library articles were edited, with new content developed relevant to depression, lifestyle and pregnancy. The voiceover for animations was replaced with antenatal scenarios. Content of many practice change activities and examples were changed to reflect the concerns of antenatal women. The partner support website was edited to be antenatal-focused. No changes were made to the core CBT components and the structure of the program itself. The low intensity telephone coaching component was retained as it was a strongly endorsed feature of the postnatal program. Rather than providing therapy per se, the support provided by the telephone coach is intended to encourage women to use the program, monitor progress, reinforce gains and answer questions [16, 25, 26].

### Feasibility Trial

#### Participants

The study was advertised via flyers in practices of health professionals (e.g., General Practitioners), social media posts, and an advertisement on the Ovia Pregnancy smartphone application, which offers educational content for pregnant women [27]. All women completed the Edinburgh Postnatal Depression Scale (EPDS: [15]) and were included in the study if they satisfied eligibility criteria.

*Inclusion Criteria*: pregnant and an EPDS score of 11–22 (inclusive).

*Exclusion criteria*: living outside Australia, < 18 years old, current diagnosis of substance abuse, bipolar disorder, post-traumatic stress disorder (PTSD) or depression with psychotic features meeting DSM-IV criteria [28]; moderate to high risk of suicide; current depression treatment (psychotherapy or antidepressant treatment).

A sample size of 26, based on replicating the large effect sizes found in the postnatal version of this program, provided sufficient power (0.80) to detect large effect sizes at α = 0.05 [29].

### Measures

*SCID-IV:* The Structured Clinical Interview for DSM-IV (SCID-IV) was conducted by psychologists over the phone to assess eligibility [28]. The SCID-IV was purchased for use by the Parent-Infant Research Institute.

*PHQ-9:* The Patient Health Questionnaire (PHQ-9: [30], is a measure of depressive symptoms within the last 2 weeks and has been validated for use in antenatal populations [31], was delivered by telephone at baseline, week 3, week 5, week 8 and 3 months postpartum to monitor participant safety and measure symptom change. The PHQ-9 is in the public domain and no permission was required for use.

*DASS-21:* Depression, anxiety, and stress symptom severity was measured by the 21-item Depression Anxiety Stress Scales: DASS-21 [32] as part of the baseline, week 8 and 3 months postpartum online questionnaires, sent in a link via SMS to participants’ mobile phones. The DASS has been validated for use in antenatal populations [33]. The DASS-21 is in the public domain and no permission was required for use.

### Program acceptability

Features of the online program and coach calls were rated on 4-point Likert scales from ‘not at all helpful/satisfied’ to ‘very helpful/satisfied’ as part of the week 8 questionnaire.

### Procedure

Prospective participants expressed interest online, and then completed the telephone assessment to determine eligibility and complete their baseline PHQ-9. If eligibility criteria were met and consent forms returned, they were then sent a link via SMS to a Google Form containing the baseline (pre-intervention) questionnaires, which included the DASS-21. After completing baseline questionnaires, participants were reimbursed $30 for their time and enrolled as a user. They received a ‘welcome call’ from a coach to encourage them to begin using the program and to schedule their first coach call one week later.

Participants worked through the *Mum2BMoodBooster* program and received weekly phone calls from a coach (psychologist or provisional psychologist). During these low-intensity telephone support calls (maximum 30 min per week), coaches reinforced progress made, encouraged use of the program and the practice of strategies, and introduced upcoming sessions. Coaches had access to each participant’s program usage via an administrative website in order to tailor their support. Coaches followed session guides during their calls. There were eight coaches in the study and one personal coach was allocated per participant. However, a small number of participants (*n* = 4) received calls from more than one coach due to staff leave.

Participants received questionnaires via a link in an SMS to a Google Form in week 8 and 3 months postpartum. Participants were reimbursed AUS$20 for completion of each follow-up questionnaire.

### Statistical Analysis

Missing data were not included in analysis; all results are from observed-case analyses. Changes in mental health outcome scores were analysed by paired t-tests. This was the approach used in the feasibility trial of our postnatal MumMoodBooster and the same procedure was used here so that effect sizes would be directly comparable with that trial. As difference scores between time-points were required for the subsequent calculation of t-tests, only cases with data at both time-points (baseline and week 8, or baseline and 3 months postpartum) were used. Visual inspection Q-Q plots, Kolmogorov–Smirnov and Shapiro-Wilks tests were used to assess normality.

## Results

### Sample demographics

Of 1149 women who clicked on the study advertisement, 269 continued to the study website and completed online screening. Based on initial responses, 105 were considered for intake. Of these, 19 were ineligible (due to miscarriage/ectopic pregnancy: *n* = 5; high suicide risk: *n* = 2; PTSD: *n* = 7; complex psychological history: *n *= 1; or currently receiving treatment: *n *= 4), 22 declined at intake, and 37 could not be contacted for intake. The final sample included 27 women (one more than target sample size). At online screening, EPDS scores ranged between 11 and 22 (*M* = 16, ± 3.12). Table 1 shows participants’ SCID-IV diagnoses.

**Table 1:** Diagnostic Status of Feasibility Trial Participants at Baseline

Diagnosis	N
Current major depression & past major depression	15
Current major depression	1
Current major depression & codeine dependence	1
Current major depression & past Manic Episode	1
Current minor depression	1
Current minor depression & past major depression	2
Current bereavement & past major depression	1
No current depression diagnosis, past alcohol abuse	1
No current depression diagnosis, past major depression	2
No current or past depression diagnosis	2

At enrollment, participants ranged in age from 23–38 years (*M* = 32.19, ± 3.23), and were between 6 and 35 weeks pregnant, with 26% (7/27) in trimester one, 55.5% (15/27) in trimester two and 18.5% (5/27) in trimester three. Fifteen were pregnant with their first child, ten with their second, one with their third, and one with their fourth. Most women were from metropolitan and urban areas of Australian states and territories, with only one woman living in a rural area. Table 2 shows further demographics of the sample.

**Table 2:** Baseline Demographics of Participants in Feasibility Trial

Characteristic	N
**Country of birth**	
Australia	18
New Zealand & Oceania	1
Europe	3
Asia	1
Africa	2
South America	2
**State of residence**	
NSW	11
Victoria	10
Queensland	2
Western Australia	2
South Australia	1
Northern Territory	1
**Relationship status**	
Married/De Facto	23
Single	3
Uncertain	1
**Education**	
Bachelor or postgraduate degree	10
Advanced diploma/ certificate	10
Certificate level/apprenticeship	4
High School (year 12)	2
Did not finish school	1
**Family Income ($AUD)**	
20,001–60,000	3
≥ 80,001	22
Not disclosed	2

### Treatment adherence and program usage.

**Fig. 1:**
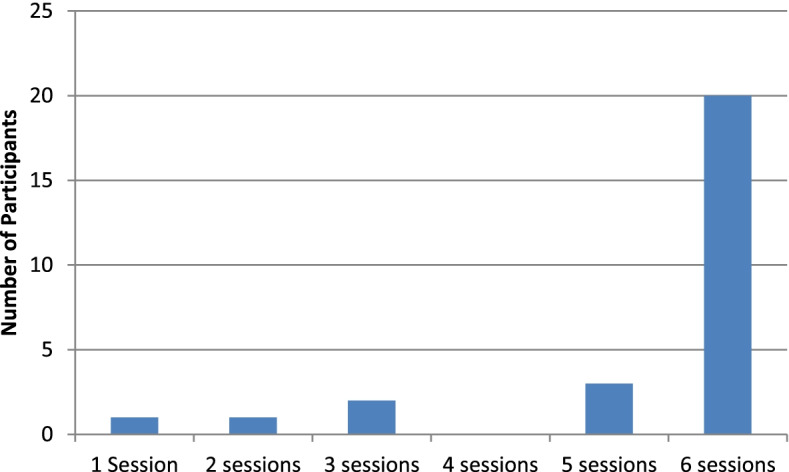
Number of *Mum2BMoodBooster* sessions completed

On average, participants visited 5.33 (± 1.39) *Mum2BMoodBooster* sessions out of 6. Figure 1 shows the majority (74%) visited all 6 sessions. Seventy percent (*n* = 19) also participated in all 6 coach calls, with the majority completing their sixth coach call within 7 weeks of commencing the program (range = 6–15 weeks). One participant delivered her baby prior to the post-treatment time point, and engaged with the program over 15 weeks. On average, participants spent 220 (± 118) minutes in the program, across an average of 13.70 (± 7.14) individual visits, averaging 16.84 (± 6.53) minutes per visit.

The average number of distinct activities visited (out of 63) was 16.44 (± 9.01) and the average number of distinct library articles read (out of 9) was 2.30 (± 2.02). Table 3 details the popularity of each library article. Partners of approximately half of the participants accessed the partner support website (13/27).

**Table 3:** Library article and times accessed

Article Title	Number of participants who accessed
Communication Skills	11
Solving Problems	7
Getting Support	8
Sleep & Caring for Baby	4
Managing Your Stress	10
Your Baby’s Needs	3
Managing Your Time	8
You & Your Partner	11

### Mental health outcomes

Tables 4 and 5 show baseline, week 8, and 3 month postpartum scores for the DASS-21 and the PHQ-9.

**Table 4:** Pre-intervention, week 8, and difference scores

		**Baseline (Pre-Intervention)**		**Post-Intervention (week 8)**		**Pre/Post-intervention (week 8) Difference**			
	**N**	**Mean**	**SD**	**Mean**	**SD**	**Mean**	**SD**	**CI lower**	**CI upper**
**Depression Score**^**a**^	19	15.05	6.05	7.38	6.99	-7.68*	6.23	-4.68	-10.69
**Anxiety Score**^**b**^	19	6.95	4.78	4.63	3.76	-2.42	5.32	0.14	-4.98
**Stress Score**^**c**^	19	17.37	7.46	15.37	8.62	-2.00	9.31	2.49	-6.49
**PHQ-9 Score**^**d**^	16	13.00	4.98	5.63	2.94	-7.38*	4.32	-5.07	-9.68

^*^ *p* < .0001. ^a^DASS-21 Depression score: Normal 0–9, Mild 10–13, Moderate 14–20, Severe 21–27, Extremely Severe 28 + . ^b^DASS-21 Anxiety score: Normal 0–7, Mild 8–9, Moderate 10–14, Severe 15–19, Extremely Severe 20 + . ^**c**^DASS-21 Stress score: Normal 0–14, Mild 15–18, Moderate 19–25, Severe 26–33, Extremely Severe 34 + . ^d^PHQ-9 score: Minimal or none 0–4, Mild 5–9, Moderate 10–14, Moderately severe 15–19, Severe 20 +

**Table 5:** Pre-intervention, 3-months post-partum, and difference scores

		**Baseline (Pre-intervention)**		**Post-intervention (3 months post-partum)**		**Pre/Post-intervention Difference**			
	**N**	**Mean**	**SD**	**Mean**	**SD**	**Mean**	**SD**	**CI lower**	**CI upper**
**Depression Score**^**a**^	23	14.43	6.85	6.17	7.43	-8.26*	6.30	-5.53	-10.99
**Anxiety Score**^**b**^	23	6.70	4.77	3.13	3.66	-3.57*	5.43	-1.22	-5.91
**Stress Score**^**c**^	23	17.57	7.41	13.57	9.04	-4.00	11.52	.98	-8.98
**PHQ-9 Score**^**d**^	21	13.86	4.89	4.67	4.50	-9.19*	5.95	-6.48	-11.90

^*^ *p* < .005. ^a^DASS-21 Depression score: Normal 0–9, Mild 10–13, Moderate 14–20, Severe 21–27, Extremely

Severe 28 + . ^b^DASS-21 Anxiety score: Normal 0–7, Mild 8–9, Moderate 10–14, Severe 15–19, Extremely Severe 20 + . ^c^DASS-21 Stress score: Normal 0–14, Mild 15–18, Moderate 19–25, Severe 26–33, Extremely Severe 34 + . ^d’^PHQ-9 score: Minimal or none 0–4, Mild 5–9, Moderate 10–14, Moderately severe 15–19, Severe 20 +

Paired-samples t-tests showed a significant decrease between baseline and week 8 for DASS-21 depression scores, t(18) = 5.38, *p* < 0.0001, and for PHQ-9 scores, t(15) = 6.83, *p* < 0.0001, but not for anxiety, t(18) = 1.99, *p* = 0.06, and stress, t(18) = 0.94, *p *= 0.36. At three months postpartum, a significant decrease from baseline was found for DASS-21 depression scores, t(22) = 6.28, *p* < 0.0001, and for PHQ-9 scores, t(20) = 7.07, *p* < 0.0001. A significant decrease was also found for DASS-21 anxiety scores, t(22) = 3.15, *p* = 0.005, but not for stress, t(22) = 1.67, *p* = 0.11.

**Fig. 2:**
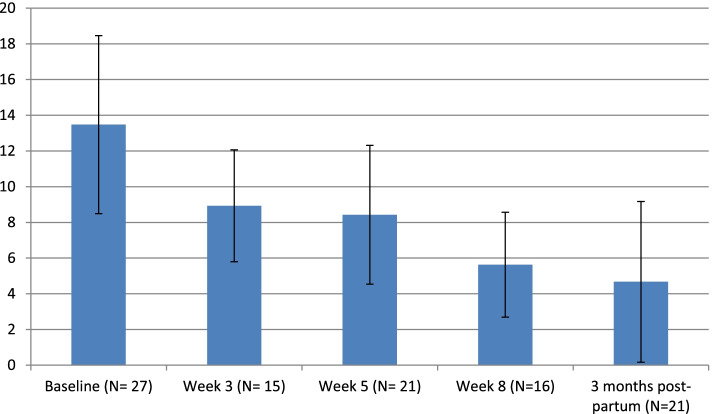
Mean PHQ-9 scores of feasibility trial participants across time (± 1SD)

Figure 2 shows the reduction of average PHQ-9 scores across the five time-points: baseline, week 3, week 5, week 8 and 3 months post birth. By week 3 the average PHQ-9 score had dropped from the moderately depressed range [10–14] to the mildly depressed range [5–9] and this was maintained at week 5 and week 8. Of the women who completed the week 8 PHQ-9, 75% were in their second trimester and 25% were in their third. By 3 months postpartum, the average PHQ-9 score dropped into the lowest range, minimal or no depression (0–4).

### Program acceptability

Table 6 shows the participants’ ratings of program features.

**Table 6:** Program acceptability (4-point Likert-type scale)

Question	*M (SD) n* = *23*	*%* of participants who responded ‘moderately’ or ‘very helpful/satisfied’
How helpful did you find the *Mum2BMoodBooster* program?	3.30 (± 0.70)	87.00
Overall, how helpful did you find the Personal Coach Calls?	3.52 (± 0.85)	87.00
How satisfied are you with the strategies for reducing negative thinking feature of the *Mum2BMoodBooster* program?	3.00 (± 0.85)	73.90
How satisfied are you with the strategies for increasing positive thinking feature of the *Mum2BMoodBooster* program?	3.00 (± 0.74)	73.90
How satisfied are you with the pleasant activities feature of the *Mum2BMoodBooster* program?	3.00 (± 0.90)	69.60
How satisfied are you with the mood tracking feature of the *Mum2BMoodBooster* program?	2.78 (± 0.90)	56.50
How satisfied are you with the partner support program feature of the *Mum2BMoodBooster* program?	2.22 (± 0.90)	43.50
How satisfied are you with the library articles feature of the *Mum2BMoodBooster* program?	2.65 (± 0.93)	43.50
How satisfied are you with the videos feature of the *Mum2BMoodBooster* program?	2.61 (± 1.03)	39.10

## Discussion

This study aimed to assess the acceptability and efficacy of the *Mum2BMoodBooster* program, designed for pregnant depressed women, in a feasibility study with 27 women. The majority of women had a DSM-IV diagnosis of major depression (n = 18), a number presented with minor depression or other diagnoses, and only one woman had no diagnosis at baseline.

Post-intervention, women showed improvements in mood, as reflected by significant decreases in depression scores at week 8, which were maintained at 3-month follow-up on both the DASS-21 and PHQ-9. As in the RCT of our postnatal *MumMoodBooster* program [16], the average PHQ-9 score was reduced from the “moderately depressed” range at baseline to the “minimal or no” symptoms range. These results provide preliminary evidence for the program, and warrant further evaluation in an RCT. An easily available treatment for antenatal depression has the potential to benefit not only women, but also their children. Emerging evidence suggests that treatment of maternal mental health difficulties in pregnancy may have some beneficial impacts on early infant developmental markers [34].

The results also showed statistically significant decreases in anxiety (but not stress) as measured by the DASS-21. This was despite the relatively low level of baseline anxiety in the study sample, which averaged in the “normal” range (stress scores were in the “mild” range). Although RCTs of the postnatal program [16, 26] showed larger reductions in anxiety and stress than seen here, baseline levels of anxiety and stress were higher. Whilst anxiety levels in this study sample appeared mild, anxiety often accompanies perinatal depression, affecting 7.9% of antenatal women [35]. Wisner and colleagues [36] estimate that this represents almost two thirds of US women with depression.

Program usage was high with 74% of women visiting all six sessions and spending an average of 3.7 h in the program overall, across an average of 13.7 individual visits, only slightly lower than the postnatal version [25]. This slightly lower usage may not be entirely surprising—all but two of the women in our sample were working, which is commonplace for antenatal women in Australia, at an estimated 73% [37]. Indeed, two of the more popular library articles were ‘Managing Your Stress’ and ‘Managing Your Time’ with 10 and 8 out of 27 women reading them, respectively. Previous research indicates that stress is a common complaint for time-poor Australian mothers [38].

Interestingly, the most popular library articles were not only related to the mother’s own self-care (particularly stress management) and support, but also to the couple relationship with 11/27 participants opening the articles ‘You and Your Partner’ and ‘Communication Skills’. A similar number (13/27) accessed the partner support website. Changes to their relationship with their partner might be an important concern for antenatal women [39]. By contrast, fewer articles about caring for the baby were opened. This suggests that many women were focused more on immediate help for themselves and their relationship.

Overall, 87% of respondents at week 8 found the *Mum2BMoodBooster* program ‘moderately helpful’ and ‘very helpful’ with coach calls being the most endorsed feature, followed by strategies for reducing negative thinking and increasing positive thinking, pleasant activities, and mood tracking. This is in line with research demonstrating the importance of a coach in online therapies [40]. However, it also raises an important issue for implementing the intervention at scale. Although the coaching role could be delivered by professionals such as nurse practitioners, general practitioners and psychologists (a clinician portal has recently been added), even coaching of a low intensity nature will likely require greater resources than a purely self-guided program. It will therefore be important to balance these considerations in optimising future scale up. For example, women with more severe symptoms may benefit most from coaching support, while those with less severe symptoms may obtain a satisfactory treatment response with a self-guided format [41].

The components of the program with the lowest number of ‘moderately helpful’ or ‘very helpful’ ratings were the partner support website, library articles and videos, which are ancillary features to the core cognitive-behavioural content (negative and positive thinking, pleasant activities and mood tracking) in the program. These features could be further enhanced in future updates to the program.

Women who received coach calls for all six sessions took 6–15 weeks to complete the program. Feedback from coaches when calls were not completed as scheduled included: women reporting a lack of time to complete the online session, forgetting about their scheduled call and/or double booking their availability.

Possible limitations to interpretation of results include the nature of feasibility trials (no control group) and the heterogeneity of the sample. Although 21 women met the SCID-IV diagnostic criteria for major or minor depression, six women met criteria for other psychiatric diagnoses (e.g., substance dependency and abuse, manic episode). Thus, the program, which targets depression and anxiety, may have been insufficient for some participants. Moreover, with a feasibility study of this size, it is difficult to identify for whom the program may be most suitable. The design of future research studies might be configured explicitly to evaluate differences in outcomes for women with a current major depression diagnosis compared to those with other diagnoses with depressive features.

Stage of pregnancy also varied; at baseline women were between 6–35 weeks pregnant. Mood during pregnancy has been shown to differ across gestation with more negative affect being experienced in the first and third trimesters [42]. Also, as all participants in this feasibility trial received the treatment, there was no control group to compare the ‘active’ treatment to. At baseline, 44.5% of women were in their first or third trimesters. As low mood is commonly experienced during the first and third trimesters of pregnancy, it is possible that some of these women might have experienced fluctuation of mood over time in the absence of any treatment [42]. Despite these limitations, there was good participant retention at 3 months and the depression results, and to some extent the anxiety results, parallel those of the RCT of the postnatal *MumMoodBooster* program, which also had very little attrition at follow-up [16]. Future research might look at the possible differential effectiveness of the intervention relative to stage of pregnancy.

We aimed to collect a sample representative of the population likely to use this program; however, due to study exclusion criteria there was some homogeneity in sample demographic characteristics (the majority were partnered and in the same income bracket), and results of this small feasibility trial may not be wholly generalisable to the wider population from which participants were drawn.

## Conclusion

In conclusion, in a feasibility trial of depressed pregnant women, the *Mum2BMoodBooster* program was found to be promising as an online CBT therapy for the alleviation of low mood in pregnancy, including those with a major depressive disorder. Outcomes were similar to those reported with the postnatal version of the program in RCTs, including good acceptability and adherence [16, 25, 26]. Like the postnatal version, this therapy was delivered in conjunction with weekly (approximately 30 min) supportive telephone calls from a coach. Future research may include an RCT of this program compared to usual practice, designed to evaluate whether effectiveness varies with the severity of depression diagnosis, and might address when in pregnancy it is best to intervene.

Finally, effective, accessible internet interventions for perinatal mental health difficulties are likely to be needed more than ever, Emerging evidence suggests that the advent of COVID-19 has led to a substantial increase in mental health difficulties among perinatal women [43]. At the same time, widespread mandatory restrictions on population movements have at times been enacted in an effort to curb the progress of the COVID-19 pandemic. This means that, in many jurisdictions, access to traditional face-to-face treatments may be curtailed or problematic and digital solutions to health care are increasingly relevant.

## Data Availability

The datasets used and analysed during the current study are available from the corresponding author on reasonable request.

## Abbreviations

CBT: Cognitive behavioural therapy
DASS-21: Depression Anxiety Stress Scales
DSM-IV: Diagnostic and Statistical Manual of Mental Disorders-IV
EPDS: Edinburgh Postnatal Depression Scale
PHQ-9: Patient Heath Questionnaire
PTSD: Post-traumatic stress disorder
RCT: Randomised controlled trial
SCID-IV: Structured Clinical Interview for the DSM-IV

## References

[1] Dadi AF, Miller ER, Bisetegn TA, Mwanri L (2020). Global burden of antenatal depression and its association with adverse birth outcomes: an umbrella review. BMC Public Health.

[2] Underwood L, Waldie K, D’Souza S, Peterson ER, Morton S (2016). A review of longitudinal studies on antenatal and postnatal depression. Arch Womens Ment Health.

[3] Gavin NI, Gaynes BN, Lohr KN, Meltzer-Brody S, Gartlehner G, Swinson T (2005). Perinatal depression: a systematic review of prevalence and incidence. Obstet Gynecol.

[4] Faramarzi M, Kheirkhah F, Barat S, Cuijpers P, O'Connor E, Ghadimi R (2020). Prevalence and factors related to psychiatric symptoms in low risk pregnancy. Caspian J Intern Med.

[5] Milgrom J, Gemmill AW, Bilszta JL, Hayes B, Barnett B, Brooks J (2008). Antenatal risk factors for postnatal depression: a large prospective study. J Affect Disord.

[6] Glover V (2015). Prenatal stress and its effects on the fetus and the child: possible underlying biological mechanisms. Advances in neurobiology.

[7] Glover V (2014). Maternal depression, anxiety and stress during pregnancy and child outcome; what needs to be done. Best Pract Res Clin Obstet Gynaecol.

[8] Herba CM, Glover V, Ramchandani PG, Rondon MB (2016). Maternal depression and mental health in early childhood: an examination of underlying mechanisms in low-income and middle-income countries. Lancet Psychiatry.

[9] Waters CS, Hay DF, Simmonds JR, van Goozen SHM (2014). Antenatal depression and children’s developmental outcomes: potential mechanisms and treatment options. Eur Child Adolesc Psychiatry.

[10] O'Connor TG, Heron J, Glover V, Alspac Study T (2002). Antenatal anxiety predicts child behavioral/emotional problems independently of postnatal depression. J Am Acad Child Adolesc Psychiatry.

[11] Pearson RM, Evans J, Kounali D, Lewis G, Heron J, Ramchandani PG (2013). Maternal Depression During Pregnancy and the Postnatal Period Risks and Possible Mechanisms for Offspring Depression at Age 18 Years. JAMA Psychiat.

[12] Capron LE, Glover V, Pearson RM, Evans J, O'Connor TG, Stein A (2015). Associations of maternal and paternal antenatal mood with offspring anxiety disorder at age 18 years. J Affect Disord.

[13] Dennis C-L, Hodnett ED. Psychosocial and psychological interventions for treating postpartum depression. Cochrane Database Syst Rev. 2007;(4). Art. No.: CD006116. 10.1002/14651858.CD006116.pub2. 10.1002/14651858.CD006116.pub2 17943888

[14] Cuijpers P, Brannmark JG, van Straten A (2008). Psychological treatment of postpartum depression: a meta-analysis. J Clin Psychol.

[15] Cox J, Holden J (2003). A guide to the Edinburgh Postnatal Depression Scale (EPDS).

[16] Milgrom J, Danaher B, Holt C, Holt CJ, Seeley J, Tyler MS (2016). Internet Cognitive Behavioural Therapy for Women with Postnatal Depression: a randomised controlled trial of MumMoodBooster. J Med Internet Res.

[17] O'Mahen HA, Woodford J, McGinley J, Warren FC, Richards DA, Lynch TR (2013). Internet-based behavioral activation-Treatment for postnatal depression (Netmums): A randomized controlled trial. J Affect Disord.

[18] Pugh NE, Hadjistavropoulos HD, Dirkse D (2016). A Randomised Controlled Trial of Therapist-Assisted, Internet-Delivered Cognitive Behavior Therapy for Women with Maternal Depression. PloS one..

[19] Ashford MT, Olander EK, Ayers S (2016). Computer- or web-based interventions for perinatal mental health: A systematic review. J Affect Disord.

[20] Lau Y, Htun TP, Wong SN, Tam WSW, Klainin-Yobas P (2017). Therapist-Supported Internet-Based Cognitive Behavior Therapy for Stress, Anxiety, and Depressive Symptoms Among Postpartum Women: A Systematic Review and Meta-Analysis. J Med Internet Res..

[21] Forsell E, Bendix M, Holländare F, Szymanska von Schultz B, Nasiell J, Blomdahl-Wetterholm M (2017). Internet delivered cognitive behavior therapy for antenatal depression: A randomised controlled trial. J Affect Disord..

[22] Danaher B, Milgrom J, Seeley JR, Stuart S, Schembri C, Tyler MS (2012). Web-based Intervention for Postpartum Depression: Formative Research and Design of the MumMoodBooster Program. JMIR Research Protocols.

[23] Milgrom J, Martin PR, Negri LM (1999). Treating Postnatal Depression.

[24] Milgrom J, Negri LM, Gemmill AW, McNeil M, Martin PR (2005). A randomized controlled trial of psychological interventions for postnatal depression. Br J Clin Psychol.

[25] Danaher B, Milgrom J, Seeley J, Stuart S, Schembri C, Tyler M (2013). MumMoodBooster Web-Based Intervention for Postpartum Depression: Feasibility Trial Results. J Med Internet Res.

[26] Milgrom J, Danaher B, Seeley J, Holt C, Holt C, Ericksen J (2021). Internet and Face-to-Face Cognitive Behavioural Therapy for Postnatal Depression Compared to Treatment-As-Usual: A Randomised Controlled Trial of MumMoodBooster. J Med Internet Res..

[27] Vignato J, Landau E, Duffecy J, O'Hara MW, Segre LS (2019). Using mobile health applications for the rapid recruitment of perinatal women. Arch Womens Ment Health.

[28] First MB, Spitzer RL, Gibbon M (1996). Structured Clinical Interview for DSM-IV Axis I Disorders, Patient Edition (SCID-I/P, Version 2.0).

[29] Cohen J (1992). A power primer. Psychol Bull.

[30] Kroenke K, Spitzer RL, Williams JB (2001). The PHQ-9: validity of a brief depression severity measure. J Gen Intern Med.

[31] Sidebottom AC, Harrison PA, Godecker A, Kim H (2012). Validation of the Patient Health Questionnaire (PHQ)-9 for prenatal depression screening. Archives of Women’s Mental Health.

[32] Lovibond SH, Lovibond PF (1995). Manual for the Depression Anxiety Stress Scales.

[33] Xavier S, Bento E, Azevedo J, Marques M, Soares MJ, Freitas V, Mota D, Macedo A, Pereira AT (2016). Validation of the Depression, Anxiety and Stress Scale–DASS-21 in a community sample of Portuguese pregnant women. Eur Psychiatry.

[34] Goodman SH, Cullum KA, Dimidjian S, River LM, Kim CY (2018). Opening windows of opportunities: Evidence for interventions to prevent or treat depression in pregnant women being associated with changes in offspring's developmental trajectories of psychopathology risk. Dev Psychopathol.

[35] Falah-Hassani K, Shiri R, Dennis CL (2017). The prevalence of antenatal and postnatal co-morbid anxiety and depression: a meta-analysis. Psychol Med.

[36] Wisner KL, Sit DKY, McShea MC, Rizzo DM, Zoretich RA, Hughes CL (2013). Onset timing, thoughts of self-harm, and diagnoses in postpartum women with screen-positive depression findings. JAMA Psychiat.

[37] Australian Bureau of Statistics (2017). Pregnancy and Employment Transistions.

[38] Rose J (2017). Never enough hours in the day: Employed mothers' perceptions of time pressure. Australian Social Policy Association.

[39] Figueiredo B, Conde A (2015). First- and second-time parents’ couple relationship: from pregnancy to second year postpartum. Family Science.

[40] Mohr DC, Cuijpers P, Lehman K (2011). Supportive accountability: a model for providing human support to enhance adherence to eHealth interventions. J Med Internet Res..

[41] Karyotaki E, Efthimiou O, Miguel C (2021). Internet-Based Cognitive Behavioral Therapy for Depression: A Systematic Review and Individual Patient Data Network Meta-analysis. JAMA Psychiat.

[42] Amiel Castro RT, Anderman CP, Glover V, O'Connor TG, Ehlert U, Kammerer M (2017). Associated symptoms of depression: patterns of change during pregnancy. Archives of Womens Mental Health.

[43] Berthelot N, Lemieux R, Garon-Bissonnette J, Drouin-Maziade C, Martel É, Maziade M (2020). Uptrend in distress and psychiatric symptomatology in pregnant women during the coronavirus disease 2019 pandemic. Acta Obstet Gynecol Scand.

